# The effects of cow dominance on the use of a mechanical brush

**DOI:** 10.1038/s41598-021-02283-2

**Published:** 2021-11-26

**Authors:** Borbala Foris, Benjamin Lecorps, Joseph Krahn, Daniel M. Weary, Marina A. G. von Keyserlingk

**Affiliations:** Animal Welfare Program, Faculty of Land and Food Systems, The University of British Columbia, 2357 Main Mall, Vancouver, BC V6T 1Z6 Canada

**Keywords:** Zoology, Animal behaviour

## Abstract

An animal’s social position within a group can influence its ability to perform important behaviours like eating and resting, but little is known about how social position affects the ability to express what are arguably less important but still rewarding behaviors, such as grooming. We set out to assess if dominance measured at the feeder is associated with increased use of a mechanical brush. Over a 2-year period, 161 dry cows were enrolled in a dynamically changing group of 20 individuals with access to a mechanical brush. We determined dominance using agonistic behaviors at the feeder and retrospectively analyzed brush use for the 12 most, and 12 least dominant individuals during the week before calving. Cows that were more dominant at the feeder used the brush more, especially during peak feeding times. Agonistic interactions at the brush did not differ between dominants and subordinates and were not related to brushing duration. These findings indicate that social position, calculated using competition for feed, affects mechanical brush access such that subordinates use the brush less than dominant cows independent of competition or time of day.

## Introduction

Changes in some behaviors can be used to assess health and affective states in animals^1,2^. In contrast to behaviors such as feeding and resting, grooming is typically considered to be a less urgent^3^ behavior and less essential for survival (perhaps similar to other rewarding behaviors such as play^4^). It has been suggested that these behaviors are more likely to be suppressed early on when animals become sick^5^ or otherwise experience suboptimal living conditions^6^. On commercial dairy farms, mechanical brushes are increasingly used to provide cows with self-grooming opportunities, promoting naturalistic cleaning and scratching behaviors. Cows are motivated to use a mechanical brush^7^ indicating that they find brushing rewarding. Previous research found reduced brush use in cattle with poor health^8–11^, stress after calving^12^, and after social mixing^13^, an experience known to be stressful^14,15^. However, despite the widespread use of mechanical brushes in dairy cow groups, the influence of social position on brush use is not well understood.

We propose that complex social interactions in cattle^16,17^ may influence brush use. When dairy cattle are housed in groups, individual access to resources is regulated by dominance hierarchies^18,19^, representing the overall network of dyadic dominance relationships between individuals^20^. In addition to the group-level hierarchy and dyadic relationships, dominance can be considered a trait of the individual^21,22^. Dominant cows use physical and non-physical agonistic interactions to displace subordinates, influencing time budgets and behavioral patterns, limiting access to resources, and increasing stress^23^. In nature, animals can benefit from group membership^24^, and in these cases dominance relationships reduce the need for potentially dangerous agonistic interactions^25^. However, farm animals often have little agency in group formation^24^, and high stocking rates can cause increased competition for resources^26–28^ making it difficult for subordinate animals to avoid interactions with dominants.

Previous work has reported that, when housed in a freestall barn, the majority of agonistic interactions take place in the feeding area^18,29^ (i.e., feed bunk). Although displacements also occur in other areas of the pen^30,31^, feed related competition has been validated to be a practical proxy to measure dominance in captive dairy cow groups^32^. To avoid receiving aggressive interactions, subordinate cows can use a variety of strategies, including feeding at less competitive times^19,33^. During competitive times, some cows may also make concessions, choosing to utilize an alternative resource that is currently available instead of a preferred resource (e.g., brush instead of feed). However, it is unknown how dominance affects access to less essential resources, like a brush.

The primary aim of this study was to investigate if dominance affected access to a mechanical brush in dairy cows. We also set out to compare brush use patterns of dominant and subordinate cows over the day and to determine if variation in brush use was related to direct competition for the brush. We hypothesized that (1) dominant individuals (as per their competitive behavior at the feeding area) will spend more time using the brush than subordinate individuals, (2) the number of displacements performed and received at the brush will influence individual brush usage with dominant individuals performing more and receiving fewer displacements than subordinate individuals, and (3) the pattern of brush use throughout the day will differ between dominants and subordinates.

## Material and methods

### Ethics statement

The study was carried out in compliance with the ARRIVE guidelines. Procedures were approved by the University of British Columbia Animal Care Committee (Protocol A14–0040). Animals were cared for and procedures were performed following the Canadian Council on Animal Care Guidelines on the Care and Use of Farm Animals in Research, Teaching and Testing^34^.

### Animals and housing

The cows used for this research were part of a larger 2-year study^35^. Pregnant non-lactating cows were kept in a dynamically changing group of 20 individuals from 3 weeks before expected calving date to the first signs of calving (i.e., udder enlargement, milk letdown, relaxation of tail ligaments); cows were then moved to a separate maternity pen. The prepartum pen was equipped with 24 sand-bedded lying stalls, 2 electronic water troughs and 12 electronic feed bins (Insentec RIC System, Marknessee, The Netherlands). A mechanical brush (Luna, Lely, Maassluis, The Netherlands; Supplementary Video 1) was placed in the feeding alley, opposite to the feed bunk. Cows were fed in a competitive environment, comparable to common practices on dairy farms; all 20 individuals had access to 12 feed bins and fresh feed (total mixed ration consisting of 39% corn silage, 28% grass silage, 9% alfalfa hay, and 24% concentrate and mineral mix), which was delivered twice daily at approximately 8 h and 16 h. For each individual visit to a feed or water bin, the start and end times along with the cow identity were recorded electronically using radiofrequency identification of ear tags.

### Dominance

Agonistic ‘replacements’ at the feed and water bins (i.e., one cow pushing another cow away from a bin and then occupying the same bin; Supplementary Video 2) were detected using electronic visit data. A short interval (26 s or less) between the subsequent visits of two cows at one bin has been validated to reflect agonistic replacements; these can be detected automatically using the algorithm developed by Huzzey et al.^36^ and modified by Foris et al.^32^. Dominance was calculated using replacements, previously validated to represent the hierarchy of the animals in the pen^32^.

We calculated dominance scores to capture the competitive characteristics of individual cows living in a dynamic social environment. To enable continuous dominance calculations in a dynamic group, we used the Elo-rating^37^ (R package *EloRating*) to assign dominance scores based on the temporal sequence of agonistic interactions. Each cow started with a pre-assigned score (1000 points) when entering the prepartum group. After each replacement at the bins, both individual scores were updated: the actor (cow that initiated the replacement; winner) gained points and the reactor (cow that was replaced; loser) lost points. The scores were updated based on Eqs. (1) and (2) adapted from Neumann et al.^37^:

1 $$WinnerRating_{new}=WinnerRating_{old}+ \left(1-p\right)\times k$$

2 $$LoserRating_{new}=LoserRating_{old}- \left(1-p\right)\times k$$

The variable ‘*p*’ represents the expectation that the actor would replace the reactor cow and is calculated using the absolute difference in the scores of the cows prior to the interaction^37–39^. The constant ‘*k*’ (the maximum number of points that could be gained or lost at each replacement) was set to 20 to account for the high daily number of interactions in the group and to avoid single replacements having a disproportionally large effect on the scores. For example, if one cow replaced another with the same Elo-rating then the actor gained 10 points and the reactor lost 10 points. Expected outcomes (i.e., a dominant cow replacing a subordinate cow) caused 0–9 points to be gained and lost, while unexpected outcomes (i.e., a subordinate cow replacing a dominant cow) caused 11–20 points to be gained and lost, the number of points being proportional to the difference between actor and reactor scores prior to the interaction. The Elo-rating was calculated and updated daily for each cow throughout the duration of the trial.

**Figure 1:**
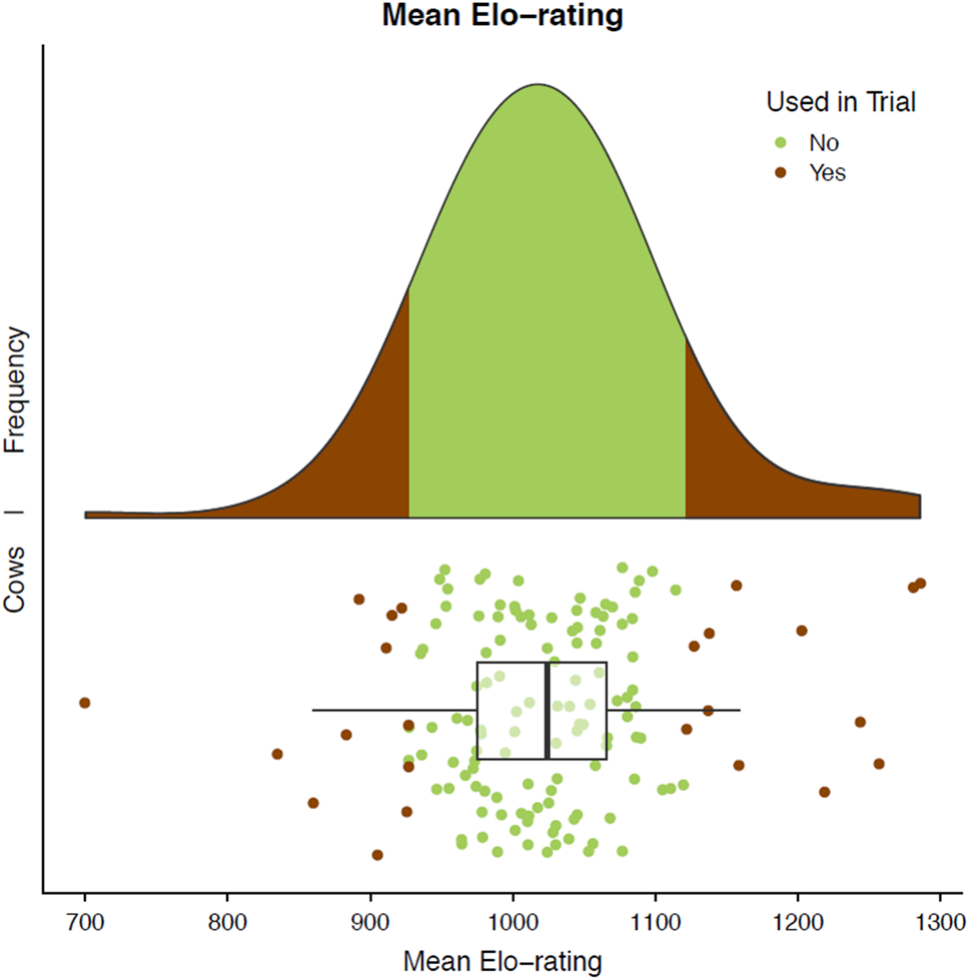
Dominance distribution shown as mean Elo-ratings calculated the 7 days before calving for multiparous cows (n = 161) housed in a dynamic prepartum group of 20 individuals monitored for 2 years. The 12 most and 12 least dominant cows with available video data and no health issues (shown with brown) were analyzed for brush use.

Of the 337 cows originally described by Neave et al.^35^, we selected 161 multiparous cows (parity mean ± SD: 3.4 ± 1.7) and determined their mean Elo-rating for the week before calving. We focused on cows with an Elo-rating below the 0.15 and above the 0.85 percentile and retained only those cows with available video data on brush use (n = 25), excluded a cow with known health events (n = 1), and analyzed the brush use for the remaining 12 most dominant and the 12 most subordinate cows (Fig. 1).

### Brush use

Video recordings (WV-CW504SP, Panasonic, Osaka, Japan) were used to retrospectively assess brush use of the 24 focal cows. Cows had been marked with hair dye to enable identification in the video. Three video observers (intra- and inter-rater intraclass correlation coefficients: ICC > 0.95) followed each cow during the 7 days before calving. Using continuous event sampling, the observers recorded brush use and all events in which the focal cow performed or received a displacement at the brush. A brush use event was recorded if a cow had physical contact with the brush and pushed it to activate the rotation function or actively rubbed herself against the brush without it starting to rotate. A displacement was defined as the performer having physical contact (headbutt or push) with the recipient cow while she was using the brush, resulting in the recipient leaving and the performer starting to use the brush.

### Statistical analysis

First, we calculated the daily brush use duration for each cow and used the ICC to determine the consistency of brush use over 7 days.

Second, we investigated how brush use duration related to dominance at the feed bunk, and the number of performed and received displacements at the brush. Due to the low daily number of displacements at the brush, total weekly data were analyzed. Weekly brush use was examined using 3 separate linear models containing either dominance status (two levels: dominant, subordinate) or the total number of performed or received brush displacements as the independent variable. In addition, we used 2 linear models to assess the effect of dominance (two levels: dominant, subordinate) on the weekly number of performed and received displacements at the brush. This analysis was completed in R (version 3.5.3, R Foundation for Statistical Computing, Vienna, Austria), with the significance set to *P* < 0.05.

Third, we investigated differences in the brush use patterns of dominant and subordinate cows over the day (this analysis completed in SAS version 9.4; SAS Institute Inc., Cary, NC). We determined mean daily brush use duration for 3-h periods, starting at midnight for each cow. In a similar manner, we calculated the mean time spent at the feed bunk for each cow based on electronic bin data. We used separate linear models to investigate the effect of period, dominance, and the interaction between the two on (1) brush use duration and (2) the time spent at the feed bunk.

## Results

The subordinate cows performed on average 0.68 ± 0.16 replacements per replacement received, versus an average of 2.26 ± 1.04 performed replacements per replacement received for the dominant cows. On average, cows used the brush for 27.4 min per day with considerable variability between individuals (SD: 21 min, range: 0–101.9 min). The daily brush use duration of cows was repeatable with most of the variation explained by differences between individuals (ICC = 0.75).

Dominant cows used the brush almost two and a half times longer than the subordinate cows (mean ± SD: dominants: 270 ± 140 min/week, subordinates: 114 ± 49 min/week; F_1,22_ = 12.84, *P* < 0.002). The number of displacements performed or received at the brush did not influence the total weekly brush use duration (Supplementary Fig. 1; actor: F_1,22_ = 1.76, *P* = 0.198, reactor: F_1,22_ = 0.098, *P* = 0.757). Dominant and subordinate cows did not differ in the number of displacements performed or received at the brush (Supplementary Fig. 1; actor: F_1,22_ = 2.22, *P* = 0.15, reactor: F_1,22_ = 1.90, *P* = 0.182).

**Figure 2:**
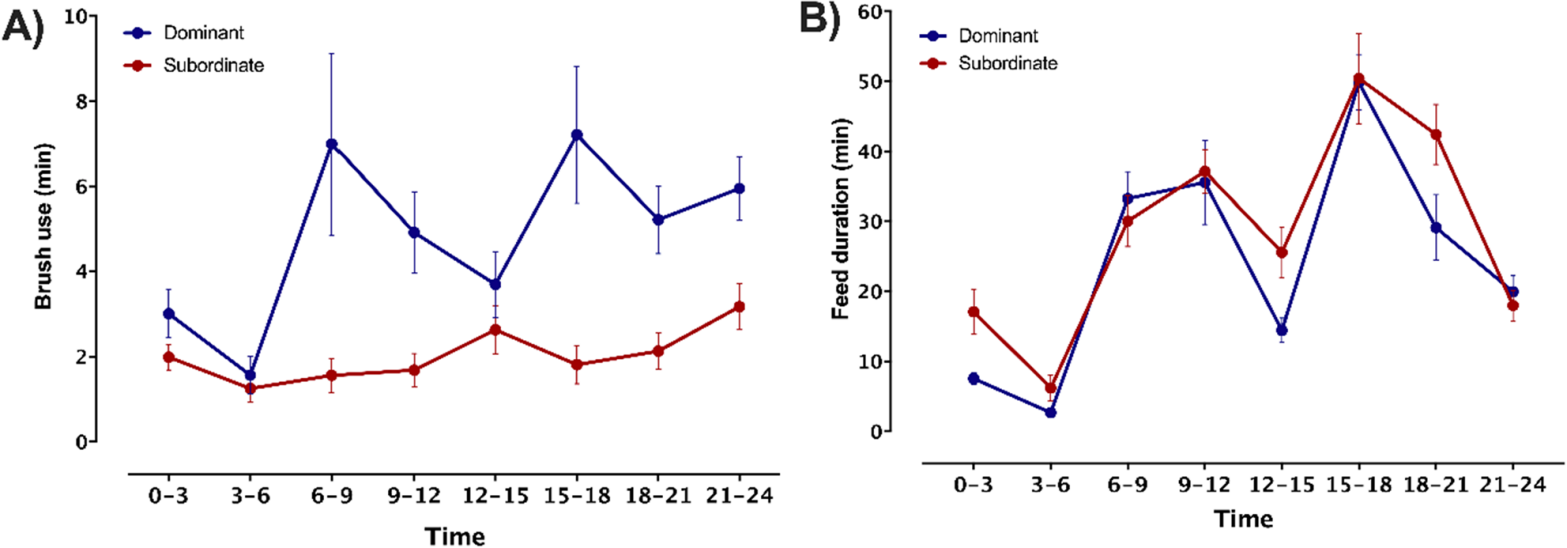
Mean brush use time (**A**) and time spent at the feed bunk (**B**) during 3-h periods of the day for dominant (n = 12) and subordinate (n = 12) cows during the week before calving.

Brush use varied over the day for dominant cows, but subordinate brush use remained low regardless of period (period x dominance interaction: F_7,154_ = 3.51, *P* = 0.0016; Fig. 2A); compared to other times of the day, dominant cows used the brush more around feeding times (i.e., 6–9 h and 15–18 h). We observed no difference between dominance categories in the temporal pattern of time spent feeding (i.e., no period × dominance interaction; F_7,154_ = 1.48, *P* = 0.178; Fig. 2B).

## Discussion

The average daily brush use in our study was similar to brush use reported for prepartum (non-lactating) cows^40^ (31.5 min/day) but higher than the 2–3 min/day^9^ or 5–7 min/day^41^ reported for lactating cows. Differences in the lactation status of cows and management differences (i.e., the milking and feeding routine) may explain this variability between studies. Differences between studies in the cow-to-brush ratio may also contribute this variability. We found that brush use duration was consistent across days within individuals. Similarly, some consistency in daily brush use was reported in Holstein calves over a 5-week period^42^ and heifers with continuous access to stationary brushes showed stable brush use levels over 6 days^43^. Previous work reported considerable individual variability in brushing behavior when cows are group housed^8^, but did not attempt to explain these differences. Based on the results of this study we suggest that between-cow variation in brushing behavior could be explained by differences in dominance. The high between-cow variation in brush use in our study is explained primarily by the dominant cows using the brush more than subordinates. It should be considered that the effect of dominance was likely amplified by the selection of the most dominant and subordinate cows from a larger pool of individuals. Previous studies found that dairy cow groups have a considerable percentage of bi-directional agonistic relationships^18,19^ and intransitive triads^19^ (i.e., cow A dominates B, B dominates C, but C dominates A), often leading to flat hierarchies with little variation in dominance for members of the same group. These flat hierarchies are often reported when competition for an essential resource is used to assess dominance. We only recorded successful replacements and could not take unsuccessful displacement attempts into account. Previous work showed that most agonistic encounters at the feed bunk are replacements^32^ and unsuccessful displacements are less common. Unsuccessful displacements can be related to cows successfully defending the resource when challenged by a more subordinate animal. Some of the replacements in our data may not be aligned with dominance relationships, particularly if a dominant recipient was approaching satiation and thus lacked motivation to defend the resource. Recording only replacements may have increased the bi-directionality of agonistic relationships in our dataset and reduced the calculated dominance differences between individuals in a group. However, dominant cows had a three-fold higher proportion of replacements as actor to replacements as reactor, compared to subordinate cows, reflecting clear differences in dominance between categories.

In this study, 20 cows had access to one brush, a cow-to-brush ratio that is low by commercial standards given that many farms employ much larger group sizes^44^. Accordingly, the number of agonistic interactions at the brush was low compared to that observed at the feed and water bins, and the brush was only used 4–5 h/day by the entire group of cows. In line with previous work, displacements at the brush were not related to social status at the feed bunk^29^. Brush displacements did not influence total time spent brushing, indicating that direct physical interactions were not important in regulating brush access. In theory, subordinate animals could be unsuccessful when trying to displace dominants from the brush but, due to the physical characteristics of the brush, unsuccessful displacement attempts at the brush were rare with some cows regaining access shortly after being displaced. However, subordinate cows may have avoided the brush area due to non-physical interactions^25^ (e.g. threats) or simply due to the near presence of dominant cows^45^, explaining the lower brush use by subordinate cows at times when dominants were nearby.

The greatest difference in brush use between dominant and subordinate cows occurred around times of peak feeding activity. Although dominant and subordinate cows had similar feeding durations during peak hours, dominant cows used the brush more during these periods. Subordinate cows did not use the brush instead of feeding during highly competitive feeding times. We suggest that, during peak times subordinate cows focused their efforts on gaining and maintaining access to fresh feed. Although motivation to access a mechanical brush is similar to that for fresh feed^7^, when access to both resources is limited feeding is likely to take priority. In a competitive environment, dominant cows were likely able to feed when and where they chose while also interspersing brushing events, as they were able to easily replace a cow to regain access to feed. Subordinate cows may adopt different strategies to gain access to fresh feed, such as attentively waiting to eat until a feed bin becomes available. The quality of feed declines in the hours after fresh feed delivery due in part to feed sorting^46,47^. The longer time spent feeding by subordinate cows may be explained by these cows having to spend more time seeking nutrient dense concentrate particles in feed that has already been sorted by the dominant cows^47^. Extra feeding time may reduce the time available for subordinate cows to use the brush. However, our observations indicate that brush use durations were considerably shorter for the subordinate cows, despite having free time (i.e., time not spent lying, feeding, or drinking), making it unlikely that feeding time per se is a limiting factor. More work is needed to determine if reduction of free time (e.g., due to long milking duration^48^ in lactating cows) is associated with changes in brush use. Mandel et al.^8^ reported that brush use increases when positioned close to the feeding area compared to when placed further away. Future studies should assess the effects of brush location on use, especially around peak feeding times, and investigate if brush-location preference differs for cows of different social positions.

In our study, subordinate cows never reached the levels of brush use observed for the dominant cows, even during non-peak feeding times. The reluctance to use the brush during times of low competition suggests that subordinates are less motivated to use brushes, at least when placed in an area associated with frequent agonistic encounters. Previous work found reduced motivation to use the brush following regrouping in young cattle^13^ (although animals were tested individually and did not have to compete for brush access), supporting the prediction that the subordinate cows in our study experienced increased social stress when in the feed alley, perhaps explaining their reduced willingness to access the brush. Under naturalistic living conditions, dominant animals may experience more social stress than subordinates because of the greater costs associated with maintaining social position and defending resources^23^. In captivity, however, subordinates may experience more stress^49^, especially if they are unable to escape aggressive dominants.

Furthermore, the dry-period (i.e., the 45–60 day non-lactating period prior to calving) can be a stressful time for dairy cows, involving dietary and environmental changes as well as frequent social regrouping^50^, increasing the frequency of agonistic interactions^25,51^. While the effects of distress resulting from dietary and environmental changes may vary between individuals independent of dominance, the effects of social stress during the dry period may be more evident for subordinate cows. This reduced motivation to engage with the brush in subordinates may reflect anhedonia-like states^52^ (i.e., seen as a reduced interest in pleasurable activities, a phenomenon associated with persistent negative affective states). Rearing conditions common on commercial farms, such as those used for the current study, are associated with stressors that can induce negative affective states^53^ and social stressors may have a more detrimental effect on mood in subordinate animals. Further studies are needed to explore how social rank affects mood in dairy cattle. Monitoring brush use may help identify animals that are more vulnerable to stressors and are at risk of compromised welfare.

## Conclusion

Subordinate cows used a mechanical brush placed in the feed alley less than dominant cows, a difference not related to competition for the brush. Subordinate cows had the opportunity to use the brush but did not take advantage of it even during times of low competition at the feed bunk, suggesting a reduced motivation to use the brush by these animals. Our findings indicate that social rank may have important implications on the extent to which animals engage in rewarding behaviors, an important aspect of animal welfare.

## Supplementary Information

Supplementary Figure S1.

Supplementary Video S1.

Supplementary Video S2.

## Data Availability

Data and analysis code is available at https://doi.org/10.5683/SP3/YIWP7C.
