# Supplementary material for: The effects of cow dominance on the use of a mechanical brush
Source: Sci Rep. 2021 Nov 26;11:22987. doi: 10.1038/s41598-021-02283-2 (PMC8626463; doi:10.1038/s41598-021-02283-2)
Supplement: Supplementary file 1 — Supplementary Figure S1. [file 41598_2021_2283_MOESM1_ESM.docx]

**B)**

**A)**


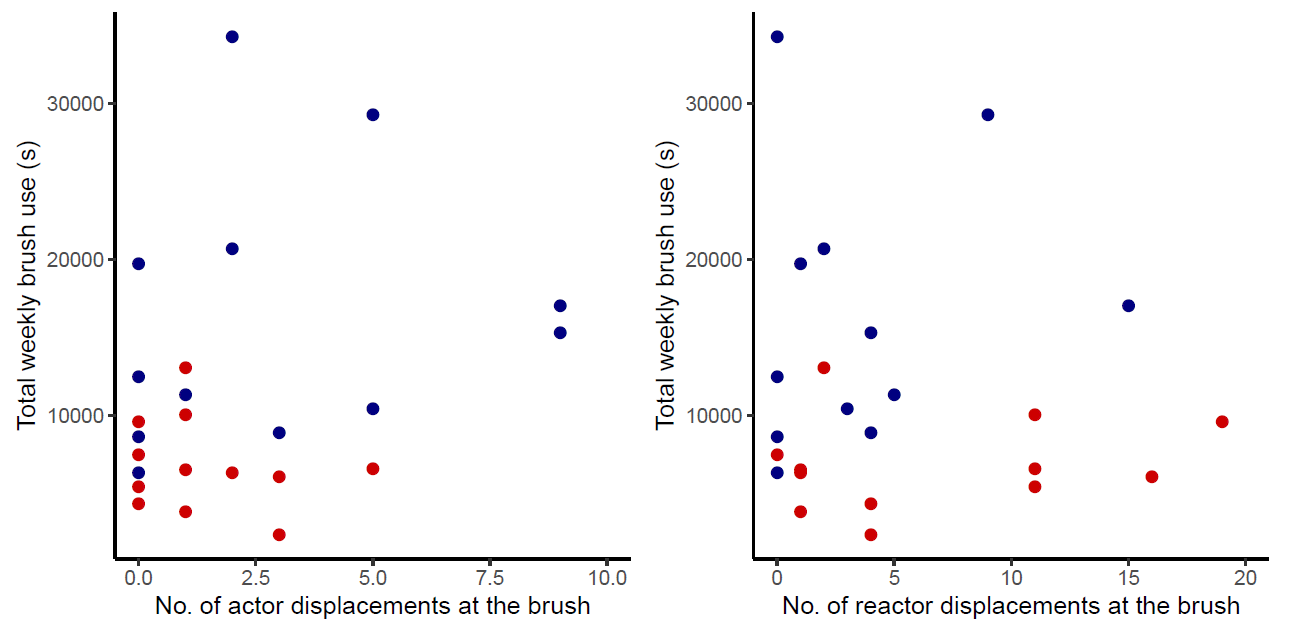


***Supplementary Figure 1.*** *Total weekly brush use of dominant (blue, n=12) and subordinate (red, n=12) cows in relation to the number of actor (A) and reactor (B) displacements at the brush. Cows were housed in a dynamic prepartum group of 20 individuals monitored for 2 years and the group had access to one mechanical brush.*
